# Subjective caregiver burden: validity of the 10-item short version of the Burden Scale for Family Caregivers BSFC-s

**DOI:** 10.1186/1471-2318-14-23

**Published:** 2014-02-20

**Authors:** Elmar Graessel, Hendrik Berth, Thomas Lichte, Hannes Grau

**Affiliations:** 1 Centre of Health Services Research in Medicine, Department of Psychiatry and Psychotherapy, Friedrich-Alexander-Universität Erlangen-Nürnberg, Schwabachanlage 6, D-91054 Erlangen, Germany; 2 Medical Psychology and Medical Sociology, Universitätsklinikum Carl Gustav Carus Dresden, Fetscherstr. 74, D-01307 Dresden, Germany; 3 Institute of General Practice and Family Medicine, Otto-von-Guericke-Universität Magdeburg, Leipziger Straße 44, D-39120 Magdeburg, Germany

**Keywords:** Subjective caregiver burden, Short version of the Burden Scale for Family Caregivers, Factor analysis, Reliability, Validity, Item difficulty, Discriminatory power

## Abstract

**Background:**

Subjective burden is a central variable describing the situation encountered by family caregivers. The 10-item short version of the Burden Scale for Family Caregivers (BSFC-short/BSFC-s) was developed to provide an economical measure of this variable. The present study examined the reliability and validity of the BSFC-s.

**Methods:**

Comprehensive data from “the IDA project” were the basis of the calculations, which included 351 dyads and examined medical data on people with dementia, interview data from their family caregivers, and health insurance data. A factor analysis was performed to explore the structure of the BSFC-s; Cronbach’s alpha was used to evaluate the internal consistency of the scale. The items were analyzed to determine the item difficulty and the discriminatory power. Construct validity was tested with five hypotheses. To establish the predictive validity of the BSFC-s, predictors of institutionalization at a follow-up time of 2.5 years were analyzed (binary logistic regression).

**Results:**

The BSFC-s score adhered to a one-factor structure. Cronbach's alpha for the complete scale was .92. A significant increase in the BSFC-s score was observed when dementia progressed, disturbing behavior occurred more frequently, care requirements increased, and when caregivers were diagnosed with depression. Caregiver burden was the second strongest predictor of institutionalization out of a total of four significant predictors.

**Conclusions:**

All hypotheses that referred to the construct validity were supported. The BSFC-short with its ten items is a very economical instrument for assessing the caregiver’s total subjective burden in a short time frame. The BSFC-s score has predictive validity for the institutionalization of people with dementia. Therefore it is an appropriate outcome measure to evaluate caregiver interventions. The scale is available for free in 20 languages (http://www.caregiver-burden.eu). This availability facilitates the comparison of international research findings.

## Background

Informal caregivers – people who provide regular care to closely related persons in need of help for a long period of time and who did not choose caregiving as an occupation – represent a large proportion of the population. Current estimates indicate that in Europe, with a population of about 750 million, about 125 million people are informal caregivers [1].

The emergence of subjective burden is a complex multivariate process. The framework of the development of the Burden Scale for Family Caregivers (BSFC) was based on two conceptual models. The Caregiver Stress Model by Pearlin and colleagues [2] describes five interacting components mediated by coping and social support: background and context (e.g. socio-economic status characteristics); primary stressors (e.g. relational deprivation); secondary role strains (e.g. job-caregiving conflict); secondary intrapsychic strains (e.g. loss of self); outcomes (e.g. physical health). The contents of these components are represented in the BSFC-s by several items. Furthermore an important aspect of subjective burden is the cognitive appraisal of these aspects according to the Transactional Stress Model by Lazarus and Folkman [3]. Stressful aspects of caregiving and its appraisal by the caregivers provided the conceptual framework for the BSFC-s.

The practical value of the theoretical concept of “subjective burden among family caregivers” has been demonstrated by the predictive power of subjective burden, which has been empirically well confirmed: At the social level of health services research, the subjective burden of family caregivers is a powerful predictor of institutionalization and, hence, of the termination of home care [4-6]).

Subjective burden, however, also affects family caregivers at the individual level, particularly with regard to health. Self-assessment reports have revealed that family caregivers show, on average, a higher number of depressive symptoms than non-caregivers but do not report significantly more physical problems [7]. Thus, caregivers and non-caregivers of the same age group do not differ in a clinically relevant manner in subjective physical health. A comparison of caregivers who were experiencing subjective burden with those who were not showed a completely different picture. The higher the subjective burden of family caregivers, the more physical symptoms were reported, e.g. in samples with more than 1,000 participants [8]. Examination of the objective criterion of mortality revealed that only caregivers who experience subjective burden are at a higher risk of mortality [9]. The mortality of caregivers who do not experience subjective burden is not significantly higher than that of non-caregivers of the same age.

The importance of subjective burden for informal care is evident in the interaction between the caregiver and care-receiver. Abusive behavior exhibited by caregivers is reported more frequently with increasing levels of subjective burden [10,11].

“Subjective burden among family caregivers” is a theoretical concept that is subject to different operationalizations. In the simplest case, caregivers are asked to assess their burden with a single-item scale. Responses are obtained with a multi-level rating scale (e.g. emotional strain by Kim and Schulz [12]). This type of burden assessment is, however, subject to limited reliability and a high risk of social desirability bias because the purpose of the survey (namely, to determine the level of burden) is apparent in how the question is phrased. For this reason, multi-item scales, which are evaluated on the basis of one or more scores, are usually used for scientific studies. The multi-factor concept of subjective burden, i.e. the use of different subscores for the different dimensions of subjective burden, has been applied, amongst others, in the Cost of Care Index [13]. This index includes five subscores for “Physical and emotional problems”, “Perception of the cared person as a provocateur”, “Personal and social restrictions”, “Economic costs”, and “Value investment in caregiving”. Another method is to compute one score that is interpreted as the “total” subjective burden. The Zarit Burden Interview [14] is based on this scoring method. The Burden Scale for Family Caregivers in the 28-item original version was developed accordingly [15].

The present study was designed to assess the validity of the 10-item short version of the BSFC, the BSFC-short/BSFC-s. It is comprised of the 10 items with the highest discriminatory power from the 28-item original version [15].

## Methods

### Design

The data used in this validation were obtained from the three-armed cluster-randomized controlled trial “the IDA project” (Dementia Care Initiative in Primary Practice) to evaluate a counseling program for family caregivers (trial registration: ISRCTN68329593) [16]. 390 people with dementia and their family caregivers were recruited by general practitioners in the study region of Middle Franconia, Bavaria, Germany [17]. Care-receivers were included if they had a physician-diagnosed primary dementia (ICD-10) and a mild or moderate cognitive impairment (Mini-Mental State Examination (MMSE) 10 to 24 points), were at least 65 years old, and were members of the AOK Bavaria - Health insurance. Care-receivers who suffered from a terminal illness or who were not able or willing to provide informed consent were excluded. Study inclusion required the signed informed consent of the care-receivers and their informal caregivers. The study was approved by the Ethics Committee at the Bavarian Chamber of Physicians (No. 05029) and was conducted in accordance with the Helsinki Declaration.

### Sample

The current sample included 351 people with dementia and their family caregivers and was obtained from an original sample that included 390 cases. 357 caregivers were available for the baseline telephone interview. There was no significant (p < .05) difference between the drop outs (n = 33) and the interviewed caregivers with regard to the data delivered by the general practitioners (characteristics of the care-receivers) and the health insurer (care level of the care-receiver, depression diagnosis of the caregiver). Caregivers were asked to maintain their consent throughout the duration of the study (n = 351). 25% of the participants were from cities with more than 100,000 citizens. The mean age of the family caregivers was 59.2 years (SD = 13.4), 73% were female, 31% were spouses, 60% were caregiving children/-in-law, and 9% were other informal caregivers. The mean age of the people with dementia was 80.3 years (SD = 6.7), and 68% were female. 64% suffered from a mild form of dementia (MMSE: 18–24 points) and 36% from a moderate form (MMSE: 10–17 points).

### Instruments

The 10 items of the **Burden Scale for Family Caregivers – short version, BSFC-short/BSFC-s** (see http://www.caregiver-burden.eu) are rated on a scale from 0 (strongly disagree) to 3 (strongly agree). The score ranges from 0 to 30 points. Higher scores indicate greater caregiver burden.

**The Mini-Mental Status Examination, MMSE**[18] is used worldwide for dementia screening. The score ranges from 0 to 30 points, with higher values indicating greater performance capacity.

The **Nurses’ Observation Scale for Geriatric Patients, NOSGER**[19] covers the most frequent aberrancies of geriatric patients as an observer rating scale. For our study, the subscales “Disturbing behavior” and “IADL” (Instrumental Activities of Daily Living) were used. Each subscale consists of 5 items rated on a scale from 1 (always) to 5 (never). The score ranges from 5 to 25 points, with higher values indicating greater impairment. The test-retest reliabilities are .84 (disturbing behavior) and .91 (IADL) [20].

The **Barthel Index, BI**[21] is an observer rating scale, which is widely used internationally to rate independence in basic activities of daily life. Higher scores indicate greater independence. Basic everyday practical capabilities are rated in 10 areas at two to four levels (0, 5, 10, 15 points). The score ranges from 0 (dependent in all areas) to 100 points (completely independent). The reliability using the Intraclass-Correlation-Coefficient (ICC) for elderly people is .89 [22].

The **Resource Utilization in Dementia – short version, RUD Lite** assesses informal care time, i.e. how many hours per day on average the primary informal caregiver provides services to the care-receiver [23]. Informal care activities are divided into three categories: ADL, IADL and supervision. While ADL includes activities such as toilet visits, bathing, and dressing, IADL comprises more complex activities such as shopping, food preparation, and housekeeping. Wimo and Nordberg [24] tested the validity. It was good for the time measurement of ADL and supervision. We used the score for total care time.

### Other measures

In addition to the measures obtained with the aforementioned instruments, we recorded the average number of **sleep interruptions** at night due to caregiving tasks or aberrancies reported by family caregivers for the last four weeks.

**Nursing care needs** were determined based on the four-level scale used in Germany to establish eligibility for nursing care benefits. This information was retrieved from the care-receivers’ long-term care insurance data. The nursing care needs were assessed by a qualified health care professional when the care-receivers applied for long-term care insurance support. The care level describes the extent to which the care-receiver is eligible to receive assistance from long-term care insurance. The classification is based on the care-receiver’s need for physical care and ranges from none (Level 0) to mild (Level 1) to moderate (Level 2) to great need for care (Level 3). For Level 3, a daily need for help of at least 5.0 hours is required (among other things). The time required is determined on the basis of standardized time corridors for certain activities.

The **depression diagnosis** was obtained from the health insurance data of the family caregivers. With regard to the feasibility of “the IDA project”, whose data form the basis of the present study, the availability of health insurance data was limited to family caregivers insured by the German health insurer AOK (168 of 351 caregivers; 48%). Presence of a diagnosis of depression was subject to the condition that a caregiver’s “depression” was classified as depression according to the ICD-10 code at least in the quarters before and after the assessment of caregiver burden. Thus, the depression diagnosis is a variable that was generated independently of all other study data, including caregiver burden.

Besides the socio-demographic data of the family caregiver and the care-receiver, the housing situation was also recorded, i.e. do the caregiver and care-receiver share a flat/house or do they live separately?

### Data recording

The general practitioners who did the recruiting collected the socio-demographic data of the care-receivers and recorded the ICD-10 diagnosis code for primary dementia as well as the MMSE score.

At baseline, trained interviewers conducted a computer-assisted telephone interview (CATI) with the informal caregivers who were primarily responsible for home care. In addition to the socio-demographic data, the caregiver burden (BSFC-s), the NOSGER subscales “Disturbing behavior” and “IADL”, as well as the Barthel Index and the RUD Lite were recorded.

The depression diagnosis at baseline was obtained from the health insurance data of the family caregiver. The care level at baseline and the outcome of “institutionalization” or “death” at a follow-up time of 2.5 years were retrieved from the care-receivers’ long-term health insurance data.

### Statistical analysis

#### Description and factor analysis

The mean, the median, the standard deviation, and the skewness of the BSFC-s score were calculated. To explore the unknown structure of the BSFC-s items, an exploratory factor analysis was performed. The distribution of the eigenvalues of the individual components is shown in a scree plot. If more than one component with an eigenvalue greater than 1.0 was obtained, orthogonal rotation would be used to obtain a simple structure of the variable grouping. The criterion for assigning a variable to a factor was defined as a factor loading ≥ .50.

#### Reliability and item analysis

Cronbach’s alpha was computed as a measure of internal consistency. Bortz and Döring [25] recommend an alpha of .80 or higher for well-designed scales. The difficulty index and discriminatory power were calculated at the item level. Because a 4-step response format (0 to 3 points) is used for the items of the BSFC-s, the ratio of the sum of the squared subject’s points to the sum of the squared item maximum ($\frac{\sum x^{2}}{\sum x_{\text{max}}^{2}}$) [26] was used to compute the difficulty index. For this index, a corridor from .20 to .80 is recommended by Bortz and Döring [25]. Discriminatory power was calculated as the deleted item-total correlation. According to Bortz and Döring, a discriminatory power of .30 to .50 is rated as moderate and a power of > .50 as high.

#### Validity

The following hypotheses (H) were tested with regard to the construct validity (H1 – H5) and predictive validity (H6) of the BSFC-s:

H1: Caregiver burden will be positively correlated with the severity of the cognitive impairment assessed by the MMSE.

H2: Disturbing behavior (operationalized using the NOSGER subscale “Disturbing behavior”) will be among the most stressful symptoms associated with dementia. Caregiver burden will be positively correlated with the severity of the disturbing behavior assessed by the NOSGER subscale “Disturbing behavior”.

H3: Caregiver burden will be associated with the mental health of family caregivers, i.e. family caregivers diagnosed with a “depressive episode” will score higher on caregiver burden than caregivers without this diagnosis.

H4: Family caregivers who have only limited possibilities to retreat will report a higher caregiver burden, i.e. caregivers sharing a flat/house with the care-receiver will have a higher caregiver burden than those living separately.

H5: The more demanding the care requirements, i.e.

–the higher the care level,

–the lower the degree of independence of the care-receiver (measured with the Barthel Index),

–the more caregiving tasks are performed at night,

–the higher the average hours of daily support, the higher the caregiver burden will be.

H6: The higher the caregiver burden, the more likely a future institutionalization of the care-receiver will be.

For testing H1 through H5, correlations between the BSFC-s score and metric variables were computed using Spearman’s rank correlation coefficient (r_S_), whereas eta was calculated to identify correlations with nominally scaled variables. Differences in the median values were tested using the Mann–Whitney or Kruskal-Wallis test. Due to the left-skewed distribution of the BSFC-s score, non-parametric procedures were given priority here.

Based on the long-term health insurance data, we determined which care-receivers were institutionalized or had died at home at the follow-up time of 2.5 years. Thus, a predictive analysis of the outcome “institutionalization”, using the baseline data as potential predictors, was computed to test H6. A binary logistic regression with “institutionalization” (coded yes = 1 and no = 0) as the dependent variable was computed. For this, the outcome “death at home” was eliminated. In a first step, bivariate analyses were computed for all independent variables to identify significant correlations (p < .05) with the dependent variable “institutionalization yes/no”. In a second step, a multicollinearity analysis was computed with all significant bivariate predictors to eliminate the confounding of predictors that were correlated with each other (correlation coefficient ≥ .50). In a third step, all significant bivariate predictors that were not affected by multicollinearity were entered into the binary logistic regression model. The variance explained by the regression model was indicated by Nagelkerke’s R^2^. The significance of predictors was estimated using the Wald coefficient.

## Results

### Distribution of the BSFS-s score

**Figure 1:**
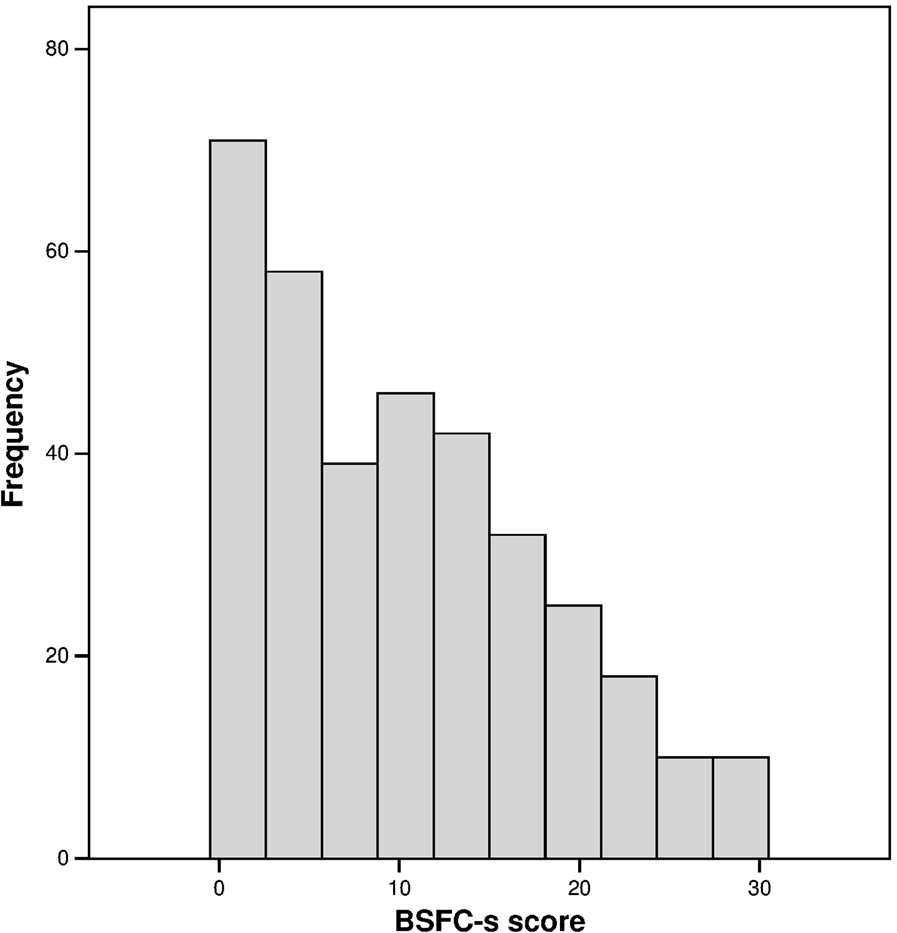
**Distribution of the BSFC-s score.** 25th percentile: 3. 50th percentile: 9. 75th percentile: 16.

The distribution of the BSFC-s score covered the entire range from 0 to 30 points (Figure 1). Due to the high frequency of low scores – the 25th percentile was 3 – the distribution was right-skewed (skewness = .57). The median was 9, and the mean was 10.2 (SD = 8.0).

### Inter-correlations of the BSFC-s items

**Figure 2:**
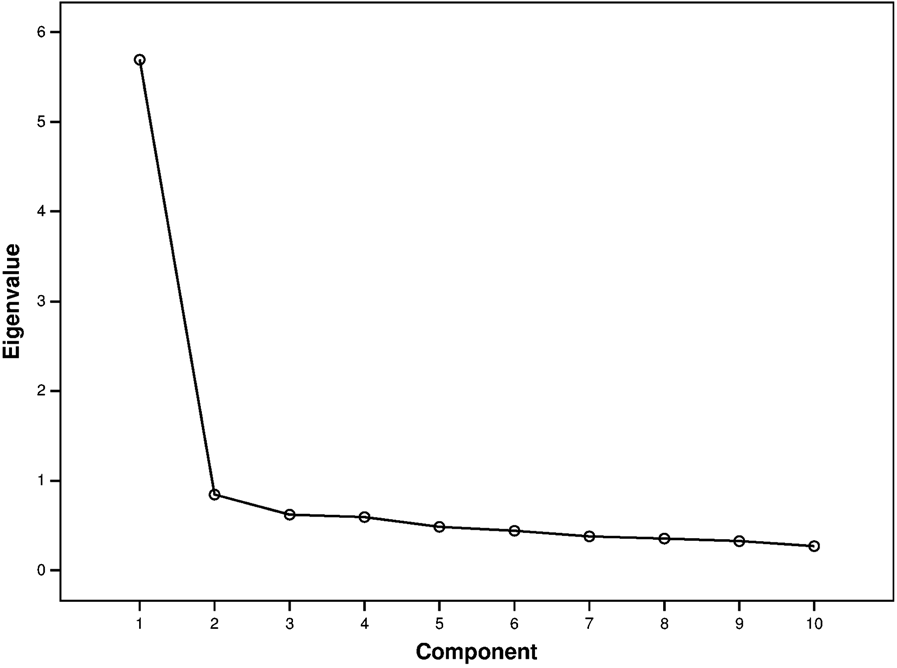
**Factor analysis of the BSFC-s score: scree plot.** Factor loadings on component 1: item 1: .81; item 2: .81; item 3: .76; item 4: .76; item 5: .81; item 6: .80; item 7: .78; item 8: .76; item 9: .62; item 10: .63.

The exploratory factor analysis yielded a 1-factor structure of the BSFC-s. Only one component had an eigenvalue greater than 1.0 (eigenvalue 5.69; Figure 2). This factor accounted for 57% of the total variance of the BSFC-s score. Each of the 10 items loaded on this factor with factor loadings that exceeded .60 (see legend Figure 2).

### Item analysis and reliability

The item difficulty of the BSFC-s items ranged from .16 to .40 (Table 1). Together with the mean item values that ranged from .7 to 1.5 (item range: 0 – 3), this finding suggests that the subjective burden of caregivers of care-receivers with mild to moderate dementia was frequently still low at the item level. All 10 items showed high discriminatory power between .55 and .75 (Table 1). For all 10 items, Cronbach’s alpha ‘if item deleted’ was below the Cronbach’s alpha that was computed for the complete scale of .915.

**Table 1:** Characteristics of the items of the BSFC-s

**Item summary**	**Mean (SD)**	**Item difficulty**	**Discriminatory power**	***P***	**Cronbach's alpha ‘if item deleted’**^**a**^
1 Reduced life satisfaction	1.1 (1.0)	.24	.74	< .001	.903
2 Physical exhaustion	1.3 (1.1)	.32	.75	< .001	.902
3 Wish to run away	1.1 (1.1)	.26	.69	< .001	.906
4 Depersonalization	.7 (1.0)	.16	.70	< .001	.906
5 Decreased standard of living	1.2 (1.2)	.30	.74	< .001	.902
6 Health affected by caregiving	.9 (1.0)	.21	.74	< .001	.903
7 Caregiving is taking strength	1.5 (1.1)	.40	.71	< .001	.904
8 Conflicting demands	1.1 (1.1)	.26	.69	< .001	.906
9 Worried about the future	.8 (1.1)	.19	.55	< .001	.914
10 Relationships with others are suffering	.7 (1.0)	.17	.55	< .001	.913

SD: standard deviation.

*P*: P value of discriminatory power.

^a^Cronbach’s alpha (10 items): .915.

### Construct validity

Caregiver burden increased significantly with the severity of the dementia syndrome; a small correlation was found (r_S_ = −.21; Table 2). The BSFC-s score was moderately correlated with the NOSGER subscale for disturbing behavior (r_S_ = .53). Caregivers who had been diagnosed with depression (n = 30; 18%) scored higher on the BSFC-s than those without a diagnosis of depression (eta = .22). Subjective burden was higher when the care-receiver and caregiver shared a flat or house than when they lived separately (eta = .19). Moderate correlations were observed between the BSFC-s score and variables that referred to the scope of caregiving: care level (eta = .31), Barthel Index (r_S_ = −.45), caregiving tasks at night (eta = .39), and number of caregiving hours per day (r_S_ = .54).

**Table 2:** Construct validity of the BSFC-s: hypotheses 1 – 5

**Hypothesis**	**Variable**	**Correlation**	***P***
**1**	MMSE	r_S_ = −.21	< .001
**2**	NOSGER Disturbing behavior	r_S_ = .53	< .001
**3**	Depression diagnosis (yes, no)^a^	eta = .22	.006
**4**	Living together (yes, no)^b^	eta = .19	.001
**5**	Care level (none, 1, 2, 3)^c^	eta = .31	< .001
**5**	Barthel Index	r_S_ = −.45	< .001
**5**	Caregiving tasks at night (0, 1, >1)^d^	eta = .39	< .001
**5**	Informal caregiving time (hours per day)	r_S_ = .54	< .001

MMSE: Mini-Mental Status Examination.

NOSGER: Nurses’ Observation Scale for Geriatric Patients.

r_S_: Spearman’s rank correlation coefficient.

Medians of the BSFC-s score for nominally or ordinally scaled variables.

(testing for median differences: Mann–Whitney or Kruskal-Wallis test).

^a^Depression diagnosis yes: median: 15.5 (n = 30; 18%).

Depression diagnosis no: median: 8.5 (n = 138; 82%).

^b^Living together yes: median: 10.0 (n = 237; 68%).

Living together no: median: 6.0 (n = 111; 32%).

^c^Care level none: median: 6.0 (n = 199; 57%).

Care level 1: median: 11.0 (n = 57; 16%).

Care level 2: median: 15.0 (n = 66; 19%).

Care level 3: median: 12.0 (n = 29; 8%).

^d^Caregiving tasks at night no: median: 6.0 (n = 229; 65%).

Caregiving tasks at night once: median: 12.0 (n = 51; 15%).

Caregiving tasks at night several times: median: 16.0 (n = 71; 20%).

All reported correlations were in the predicted direction and were significant (p = .006 was the highest p-value). Consequently, all hypotheses that referred to the construct validity of the BFSC-s were supported.

### Predictive validity

In multivariate analysis caregiver burden emerged as a significant (p = .007) and the second strongest predictor of the institutionalization of people with dementia (Table 3). Furthermore, the rate of institutionalization was significantly higher for older and more cognitively impaired people with dementia. The same applied if the caregivers did not share a flat or house with the care-receivers.

**Table 3:** Significant predictors of institutionalization at a follow-up time of 2.5 years

**Variable**	**Bivariate predictor analysis**				**Binary logistic regression**^**a**^			
	**Institutionalization yes**	**Institutionalization no**	**Test value**	***P***	**Regression coefficient B**	**Standard error**	**Wald**	***P***
	**n = 32 (12%)**	**n = 237 (88%)**						
	**n (n%) or mean (SD)**	**n (n%) or mean (SD)**						
Living situation:			Χ^2^ = 6.59	.015	−1.60	.52	9.41	.002
- together^b^	15 (8%)	165 (92%)						
- separately	17 (19%)	72 (81%)						
Caregiver burden^c^	13.7 (9.0)	9.3 (7.8)	T = −2.89	.004	.07	.03	7.38	.007
Care-receiver’s age (years)	84.4 (7.0)	79.2 (6.2)	T = −4.20	< .001	.09	.03	7.34	.007
MMSE^d^	17.4 (3.2)	19.0 (3.8)	T = 2.68	.010	-.14	.06	5.87	.015
Caregiver’s age (years)	63.1 (11.2)	58.6 (13.7)	T = −2.07	.044	.03	.02	2.57	.109
Region:			Χ^2^ = 4.56	.046	.72	.48	2.26	.133
- urban^e^	10 (21%)	38 (79%)						
- rural	21 (10%)	193 (90%)						

n = 269 cases; 351 cases of the total sample less 82 care-receivers who died at home.

^a^Chi^2^ = 41.36 (p < .001); Nagelkerke’s R^2^ = .283 (none of the 6 potential predictors had to be excluded from the multivariate analysis due to multicollinearity).

^b^Family caregiver and care-receiver with dementia share a flat or house.

^c^Score of the BSFC-s.

^d^Mini-Mental Status Examination.

^e^Urban region: cities with at least 100,000 citizens; rural region: cities with less than 100,000 citizens and villages.

Other variables were not significantly correlated with institutionalization: study arm (p = .500); sex of family caregiver (p = .527); caregiver spouse yes/no (p = .434); sex of care-receiver with dementia (p = .538); NOSGER subscale “Disturbing behavior” (p = .073); NOSGER subscale “IADL” (p = .257); family caregiver diagnosed with depression yes/no (p = .696); care level yes/no (p = .249); Barthel Index (p = .737); caregiving tasks at night yes/no (p = .532); average hours of daily care (p = .389).

The hypothesis that referred to the predictive validity of the BSFC-s was therefore supported.

## Discussion

Factor analysis of the BSFC-s revealed only one factor with an eigenvalue greater than 1.0, and all 10 items loaded on this factor with factor loadings greater than .60. Thus, empirical support was established for a one-dimensional measure of subjective caregiver burden. This was also confirmed by the high internal consistency indicated by a Cronbach’s alpha of .92 for the complete scale. Therefore it was empirically justifiable to compute a total score across the 10 items of the BSFC-s as a measure of the caregiver’s “total subjective burden”.

The reliability of the “total subjective burden” scale and, hence, the measurement precision were higher than those of their single-item counterparts [27]. The score indicates the existence and severity of a subjective caregiver burden. Thus, the urgency and necessity of imparting health services for caregivers can be derived from the BSFC-s score. On the other hand the maximum values of the single items express strong subjective burden. They indicate the aspects that constitute the individual problems of the family caregiver and in this way health services (e.g. individual counseling) can be offered as a secondary preventive or relief measure.

With only 10 items, the time required for the completion of the scale is still rather short; thus, by psychometric standards, the BSFC-s is an economical instrument. All 10 items increased the reliability of the BSFC-s score: For all 10 items, Cronbach’s alpha ’if item deleted’ was below the Cronbach’s alpha for the complete scale. All items of the BSFC-s showed high discriminatory power; the values were between .55 and .75. Item difficulty was rather low, ranging between .16 and .40. This suggests that the total subjective caregiver burden experienced by caregivers of care-receivers with mild to moderate dementia is still relatively low.

All five hypotheses that referred to the construct validity of the BSFC-s were supported. Significant but small correlations (around .20) were found for the degree of dementia symptoms, the caregiver’s opportunity to retreat, and a diagnosis of depression for family caregivers. Significant moderate correlations (greater than .50) were observed for the extent of disturbing behavior and the amount of time caregivers devoted to the home care of people with dementia. Disturbing behavior was deemed a particularly stressful condition for caregivers [28-30]. This means that the observed correlations supported the validity of the BSFC-s score.

The BSFC-s score is suitable for demonstrating that family caregivers who experience a higher subjective burden tend to terminate their caregiving activities (see the review by Luppa and colleagues [31]). After the living situation, the score at baseline was the second strongest predictor of institutionalization 2.5 years later. Therefore, the hypothesis that referred to the predictive validity of the BSFC-s could also be accepted. As only 32 cases (12%) were institutionalized at the follow-up time of 2.5 years the number of cases was limited. Thus, the influence of outliers cannot be excluded because the influence increases with a decreasing number of cases. On the other hand, previous studies had already identified subjective caregiver burden as a predictor of institutionalization (e.g. [4,32]).

In the past three decades, several scales for measuring the total subjective caregiver burden have been developed. The Zarit Burden Interview (ZBI) [14] was published first and represents the most widely used scale. The total score of the current ZBI version includes 22 items, one of which provides a global assessment of burden. The remaining 21 items are all related to the spouse/partner as the care-receiver. The German validation study by Braun and colleagues [33] was based on the interview data of 37 wives who provided care to their husbands who suffered from dementia. In contrast to the outcome of the BSFC-s, a clear single-factor structure was not found for the ZBI. Although one dominant factor was found, five other factors showed eigenvalues greater than 1 as well. Cronbach’s alpha was .91, similar to that of the BSFC-s, which had a score of .92. The average discriminatory power of the 22 items included in the ZBI was (median: .48) slightly lower than that of the BSFC-s (median: .71). Hypotheses that referred to the construct validity of the ZBI were tested using the same variables and the results were similar: Correlation with the severity of dementia (MMSE): -.26 (ZBI) vs. -.21 (BSFC-s); care requirements (Barthel Index): .68 (ZBI) vs. .53 (BSFC-s); informal care time (hours per day): .47 (ZBI) vs. .54 (BSFC-s). Consequently, with only half as many items, the BSFC-s is able to measure total caregiver burden as validly as the ZBI. The study by Braun and colleagues [31] did not analyze any predictors of institutionalization.

The strengths of the present study include the large sample size of more than 300 participants; the recruitment area, which accounts for the typical distribution of urban and rural populations; and the recruitment process, which used general practitioners to prevent bias in selecting the participants. Selection bias constitutes a major problem when participants are recruited; for example, from the outpatient departments of university hospitals. An advantage of the comprehensive IDA data is that data were combined from several sources: Specifically, medical care-receiver data, and above all, health insurance data were available in addition to the information provided by the caregivers. Moreover, the association between a self-rated depression score of caregivers and their subjective burden has been investigated before e.g. [29,34,35]. However the association between a medical diagnosis of depression in caregivers and their subjective burden was analyzed for the first time.

Due to the inclusion criteria of the underlying study, the cause for the care-receivers’ dependency on caregiving was limited to mild or moderate dementia. Another limitation of this study is that the sample was not representative of all family caregivers in Germany. The data used in this validation study were the outcome of a study (“the IDA project”) that was constructed for a purpose other than the validation of the BSFC-s. A limitation of this work is that the convergent validity cannot be verified because there was only one single measure applied to assess the subjective burden of the caregivers, the BSFC-s. Some of the data used in this validation were assessed by CATI with the family caregivers. Therefore, an observation bias by family caregivers cannot be excluded.

Further research is required to determine the test-retest reliability of the BSFC-s. Moreover, the applicability of the BSFC-s should be evaluated not only for the care of the elderly, but also for other populations such as the caretaking of children by a parent. A valid classification system denoting low, moderate, and high burden would be useful for the interpretation of the score. The extent of physical symptoms would provide a suitable criterion for this classification. The original version of the BSFC revealed a significant association between total subjective caregiver burden and the extent of physical symptoms [15].

A new study is under way to compare three different measures of the subjective burden of family caregivers: the CarerQoL questionnaire [36], the Caregiver Strain Index (CSI) [37] and the BSFC-s. Therefore, it will be possible to test for convergent validity.

A valid measure of subjective caregiver burden has important implications for both practice and research. For example, caregivers at risk of health impairments and problematic developments in the caregiving situation can be identified via screening. In this way, individual counseling could be offered as a preventive measure before caregivers suffer from overload and its consequences. Informal caregivers need timely adequate health services to maintain their resources for carrying out caregiving tasks. The implementation of effective relief measures though is important and also reasonable from a perspective of health economy [34]. The costs for the healthcare system are much higher for institutionalized care than for home care [38]. Due to its central importance for the situation of informal caregivers and caregiving in general, subjective burden is a main outcome variable for all caregiver-related interventions (see the meta-analysis by Sörensen and colleagues [39]).

## Conclusion

The study presented here shows that all hypotheses that referred to the construct validity of the BSFC-s were supported and the BSFC-s score has predictive validity for the institutionalization of people with dementia. The 10-item short version of the Burden Scale for Family Caregivers BSFC-s represents a feasible, very economical and valid short scale for measuring the caregiver’s total subjective burden. The scale is available for free in 20 languages (http://www.caregiver-burden.eu).

## Abbreviations

ADL: Activities of daily living; AOK: German health insurer [Allgemeine Ortskrankenkasse]; BI: Barthel Index; BSFC: Burden Scale for Family Caregivers; BSFC-s: Burden Scale for Family Caregivers – short version; CATI: Computer-assisted telephone interview; IADL: Instrumental activities of daily living; ICD-10: International statistical classification of diseases and related health problems - 10th revision; IDA: Dementia care initiative in primary practice [Initiative Demenzversorgung in der Allgemeinmedizin]; MMSE: Mini-Mental State Examination; NOSGER: Nurses’ Observation Scale for Geriatric Patients; rS: Spearman’s rank correlation coefficient; RUD Lite: Resource Utilization in Dementia – short version; ZBI: Zarit Burden Interview.

## Competing interests

For the data collecting original study the sponsors have commissioned two academic research institutions with the scientific evaluation of “the IDA project” by providing unconditional research funds. A contract between the sponsors and academic researchers ensures that the latter have full scientific responsibility and have the right to publish the results. Members of the sponsoring organizations closely cooperate in designing and conducting the project, but only the academic researchers have full access to all the data in this study and take complete responsibility for the integrity of the data and the accuracy of the data analysis. EG is an independent scientist who has received funding for the original study as described above. HB and HG are independent scientists and declare they have no competing interests. TL is an independent scientist and general practitioner, member of the Advisory Board of ‘CGM - Compu Group Medical’ and declares no further competing interests.

## Authors’ contributions

EG designed the original study and supervised the data collection of the original study (“the IDA project”), designed the present study and wrote the manuscript. HB gave important hints for interpretation, assisted with writing and revised the manuscript. TL gave important hints for interpretation and revised the manuscript. HG performed the statistical data analysis, gave important hints for interpretation of the data and assisted with writing the manuscript. All authors contributed to the article and have read and approved the final version of the manuscript.

## Pre-publication history

The pre-publication history for this paper can be accessed here:

http://www.biomedcentral.com/1471-2318/14/23/prepub
